# *QuickStats:* Death Rates* from Unintentional Falls^†^ Among Persons Aged ≥65 Years, by Age Group — National Vital Statistics System, United States, 1999–2020

**DOI:** 10.15585/mmwr.mm7138a4

**Published:** 2022-09-23

**Figure:**
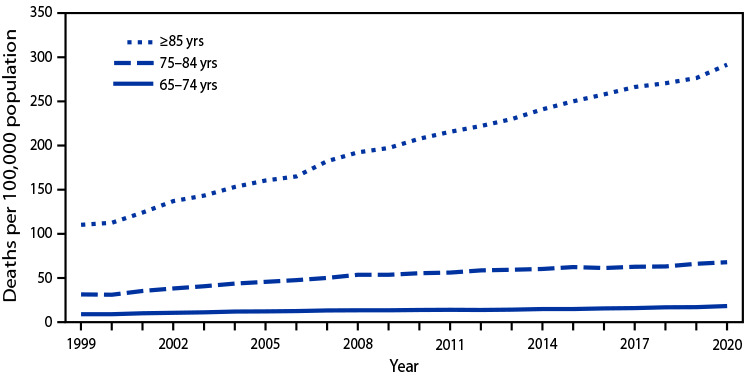
During 1999–2020, death rates from unintentional falls among persons aged ≥65 years increased among all age groups. The largest increase occurred among persons aged ≥85 years, from 110.2 per 100,000 population in 1999 to 291.5 in 2020. Among persons aged 75–84 years, the rate increased from 31.5 to 67.9, and among those aged 65–74 years, the rate increased from 9.0 to 18.2. Throughout the period, rates were highest among persons aged ≥85 years, followed by rates among persons aged 75–84 years, and were lowest among persons aged 65–74 years.

**Source:** National Vital Statistics System, Mortality Data. https://www.cdc.gov/nchs/nvss/deaths.htm

For more information on this topic, CDC recommends the following link: https://www.cdc.gov/falls

